# Influencing factors of inequity in health services utilization among the elderly in China

**DOI:** 10.1186/s12939-018-0861-6

**Published:** 2018-09-15

**Authors:** Xianzhi Fu, Nan Sun, Fei Xu, Jin Li, Qixin Tang, Junjian He, Dongdong Wang, Changqing Sun

**Affiliations:** 1 Department of Social Medicine and Health Management, College of Public Health, Zhengzhou University, 100 Kexue Avenue, Zhengzhou, 450001 Henan China; 2 Department of Management Information Systems, Terry College of Business, University of Georgia, Athens, Georgia USA; 3 College of Economics, Huazhong University of Science and Technology, Wuhan, Hubei China; 4 College of Nursing, Xinxiang Medical University, Xinxiang, 453003 Henan China

**Keywords:** Horizontal inequity, Health service utilization, Elderly, China

## Abstract

**Background:**

With the rise of the aging population, it is particularly important for health services to be used fairly and reasonably in the elderly. This study aimed to assess the present inequality and horizontal inequity for health service use among the elderly in China and to identify the main determinants associated with the disparity.

**Methods:**

This cross-sectional study was based on the sample of the survey of the China Health and Retirement Longitudinal Study (CHARLS) for 2015. The elderly was defined as individuals aged 60 and above, with a total of 7836 participants. We used the concentration index (CI) and the horizontal inequity (HI) to measure the inequity of the utilization of health services. The method of concentration index decomposition was utilized to measure the contribution of various influential factors to the overall unfairness.

**Results:**

The CI for the probability and the frequency of outpatient use were 0.1102 and 0.1015, respectively, and the corresponding values of inpatient use were 0.2777 and 0.2980, respectively. The household consumption expenditure disparity was the greatest inequality factor favoring the better-off. The Urban Employee Basic Medical Insurance made a pro-wealth contribution to inequality in frequency of health services utilization (17.58% for outpatient and 13.40% for inpatient). The contributions of New Rural Cooperative Medical Scheme on reducing unfairness in inpatient use were limited (− 2.23% for probability of inpatient use and − 5.89% for frequency of inpatient use).

**Conclusions:**

There was a strong pro-rich inequality in both the probability and the frequency of use for health services among the elderly in China. The medical insurance was not enough to address this inequity, and different medical insurance schemes had different effects on the unfairness of health service utilization.

**Electronic supplementary material:**

The online version of this article (10.1186/s12939-018-0861-6) contains supplementary material, which is available to authorized users.

## Background

The population aging is an inevitable trend in the population age structure at a certain stage of social and economic development and has now become one of the common concerns of all countries. The United Nations defines a country or region where the aging population accounts for more than 7% of the total population as an aging society [1]. While in China, the old accounted for more than 7% of the total population in 1997, signaling that China had officially stepped into an aging society. As a developing country, China is undergoing a process called “growing old before growing rich”. Moreover, the trend of aging in China is intensified. In 2015, the proportion of population aged 60 or plus in China reached 10.5% [2]. It is estimated that the absolute size and proportion of the elderly population will reach 483 million, accounting for 34.1% of the total population in 2050 [3, 4]. The challenge of population health is gradually shifting away from traditional infectious diseases to non-communicable diseases related to population aging, such as chronic diseases and disabilities [5]. Such diseases tend to bring heavy financial burden to the middle and low income elderly and exacerbate the inequity in the utilization of health services.

Equitable access to and utilization of health services is a core objective for health care systems [6]. For all individuals, health services should be provided on the principle of horizontal equality, giving priority to those who need it most [7]. In the context of population aging, it is especially important to enhance the fairness of health service utilization and promote the healthy development of the population.

In view of this, the Chinese government has implemented many targeted policies to address the issue of inequity in the utilization of health services. The establishment of the Urban Employee Basic Medical Insurance (UEBMI), New Rural Cooperative Medical Scheme (NRCMS) and Urban Residents Basic Medical Insurance (URBMI) is aimed at solving the problem of “expensive medical cost and difficult medical services” in China. With the implementation of these three kinds of health insurance schemes, the vast majority of the elderly in China were covered by medical insurance in 2011 [3]. Nonetheless, there are still great gaps in access to health services and reimbursement rates among the elderly covered by different health insurance schemes [8]. In 2009 the Chinese government initiated the “new medical reform”, and officially put forward the integration of the health insurance schemes, but UEBMI provides significantly more health benefits to insured persons than URBMI and NRCMS until now.

Several studies have investigated the influencing factors of inequity in the utilization of health services in the elderly. Cebada (2012) found that the ill-health indicators, such as comorbidities and chronic diseases, contributed greatly to pro-poor inequality in the utilization of health services [9]. Terraneo (2014) identified that the elderly with a high level of education had more resources, such as cognition, communication, relationships, which allowed them to make more informed choices and to take advantage of more health services [10].

This study, conducted from both the probability and frequency of health services utilization for the elderly in China, further determined whether the observed pattern of the inequality and horizontal inequity were patient-initiated or doctor-driven [3, 11] and sought to explore the main drivers that contribute to their inequality.

## Methods

### Data and variable

The data utilized in this study was from the survey of the China Health and Retirement Longitudinal Study (CHARLS), which was conducted by the China Centre for Economic Research of Peking University, from July to August 2015. The survey used a questionnaire to collect data in relation to demographic characteristics, socioeconomic status, health-related behaviors and lifestyles of those surveyed. Using multistage probability-proportional-to-size (PPS) sampling technique [12], a total of 21,095 individuals aged 45 years and above were sampled from 450 communities or villages in 150 counties or districts covering 12,221 households. The World Assembly on Ageing, held in Vienna in 1982, defined the elderly as those aged 60 or above [13]. Therefore, the study retained individuals aged 60 or above, a total of 8049 people. After excluding the samples with missing relevant variables, the sample size that was finally included in the study was 7836 individuals.

Four variables of health service utilization were employed. Participants were asked: (1) Have you visited a doctor for outpatient care in the last month? (2) How many times did you visit a doctor for outpatient care in the past month? (3) Have you visited a doctor for inpatient care in the previous year? (4) How many times did you visit a doctor for inpatient care in the past 12 months? The answers to questions (1) and (3) were “yes” or “no”, and the answers to questions (2) and (4) were “0, 1, 2, 3, 4, 5, etc.”

Andersen health service utilization model or behavioral model, developed in 1968, is applied internationally to research the influencing factors of health service utilization [14]. In accordance with it, independent variables were classified into three categories: (1) predisposing variables, (2) enabling variables, and (3) need variables.

The predisposing variables used as a proxy in our study were the following:gender, age, education level, marital status, and employment status.

Age was categorized into three groups: 60–69, 70–79, and 80+ years. The other predisposing variables included education level (illiterate, primary school, middle school, and high school and above), marital status (married, and unmarried), employment status (working, and no).

The enabling variables were represented by health insurance plan, pension, geographic location, and residency location. Annual per capita household consumption expenditure was also defined as a proxy of enabling variables, as household consumption expenditure is closely linked with well-being [15].

Six major types of health insurance schemes were divided into: no health insurance, UEBMI, URBMI, NRCMS, other health insurance, and two kinds of health insurance. Pensions were classified as no pension, PPGI or BPIM (Pension Program of the Government and Institutions or Basic Pension Insurance of the Firms), NRPS (New Rural Pension Scheme), old age pension allowance, other pension, and two kinds of pension. The other enabling variables included geographic location (east, central, and west), residency location (urban, and rural). Per capita household consumption expenditure was divided into five groups by the quintile method.

Additionally, need variables were represented by self-assessed health status, chronic disease, disability, disability in physical activity of daily living (PADL) and disability in instrumental activity of daily living (IADL).

Self-assessed health status was divided into very good, good, fair, poor, or very poor. A total of 14 chronic diseases (hypertension, dyslipidemia, diabetes or high blood sugar, cancer or malignant tumor, chronic lung disease, liver disease, heart attack, stroke, kidney disease, stomach or other digestive disease, emotional nervousness or psychiatric problems, memory-related disease, arthritis or rheumatism, and asthma) were included in the study. Interviewees with physical disabilities, brain damage or mental retardation, vision problem, hearing problem or speech impediment were defined as disabilities. The activity of daily living (ADL) was derived from the Activity of Daily Living Scale developed by Lawton and Brody in 1969 [16]. It is divided into the PADL (using the toilet, eating, dressing, controlling urination and defecation, getting into or out of bed, bathing or showering) and the IADL (shopping, making phone calls, cooking, doing household chores, taking medications, managing money). In the above 12 indicators, the interviewees’ inability to complete any one of the indicators independently was considered to be defined disability in PADL or IADL. Chronic diseases, disabilities, PADL and IADL were all measured as “yes” or “no”.

### Methodology

In this paper, the decision-making process of health service utilization can be divided into two phases. To some extent, the decision to see a doctor for the first time is influenced more by the patient, while the decision to repeat visits and referrals is more likely to be driven by the doctor [11]. Therefore, the study of the unfairness of the probability and frequency of health service utilization can reflect whether the observed pattern of unfairness is driven by doctors or patients, and then make specific recommendations for the results of relevant influencing factors.

The paper employed a concentration index (CI) developed by Wagstaff et al. to measure socioeconomic-related inequality in health care utilization. The CI is sensitive to the distribution of population in socioeconomic groups and ensures that the socioeconomic dimension of inequality of health services utilization is taken into account [17]. Following Wagstaff et al., the CI is defined as twice the area between the concentration curve and the diagonal (the line of absolute fairness) [18], where a concentration curve plots the cumulative percentage of use of services (y-axis) against the cumulative percentage of respondents, ranked by per capita household consumption expenditure, beginning with the least affluent and ending with the most affluent (x-axis). The concentration index ranges between [− 1,1]. When the concentration curve lies above the line of absolute fairness, the CI ranges between [− 1,0), which indicates that the disproportionate distribution of health services utilization is more concentrated among the members with lower per capita household consumption expenditure and vice versa. Zero indicates that there is no inequality.

Concentration index (CI) can be written as:

$$ \mathrm{C}=\frac{2}{\upmu}{\operatorname{cov}}_w\left({\mathrm{y}}_i,{\mathrm{r}}_i\right)\ (1) $$

where y_*i*_ is the measure of health services utilization, μ is its mean, and r_*i*_ is the relative fractional rank of an individual *i* in the distribution of per capita household consumption expenditure (*i* = 1 for the poorest and *i* = N for the richest).

The concentration index (CI) decomposing method proposed by Wagstaff reveals socioeconomic-related inequality in the health care utilization. It decomposes the CI of health service utilization into the contribution of various influencing factors to overall unfairness and provides references for the relevant suggestions of this study. Taking health services utilization as the dependent variable, it is defined in the following linear model:

$$ {\mathrm{y}}_i=\updelta +{\sum}_v{\upgamma}_{\mathrm{v}}{\mathrm{x}}_{vi}+{\sum}_j{\upgamma}_{\mathrm{j}}{\mathrm{y}}_{ji}+{\sum}_k{\upgamma}_{\mathrm{k}}{\mathrm{z}}_{ki}+{\upvarepsilon}_i\ (2) $$

Where three types of explanatory variables are identified: predisposing variables (x_*v*_) including gender, age, education, marital status, employment status, enabling variables (y_*j*_) including health insurance schemes, pension, geographic location, residency location, household consumption expenditure, and need variables (z_*k*_), such as self-assessed health status, chronic disease, disability, PADL and IADL. δ, γ and ε denote a constant, coefficient and error term respectively.

Concentration index (CI) for health service utilization can be decomposed as follow:

$$ \mathrm{C}={\sum}_{\mathrm{v}}\frac{\upgamma_v{\overline{\mathrm{x}}}_{\mathrm{v}}}{\upmu}{\mathrm{C}}_{\mathrm{y}}+{\sum}_{\mathrm{j}}\frac{\upgamma_{\mathrm{j}}{\overline{\mathrm{y}}}_{\mathrm{j}}}{\upmu}{\mathrm{C}}_{\mathrm{j}}+{\sum}_{\mathrm{k}}\frac{\upgamma_{\mathrm{k}}{\overline{\mathrm{z}}}_{\mathrm{k}}}{\upmu}{\mathrm{C}}_{\mathrm{k}}+\frac{GC_{\varepsilon }}{\upmu}\ (3) $$

Where γ_y_, γ_j_ and γ_k_ are the marginal effects of predisposing, enabling and need variables, $\overline{\mathrm{x}}$, $\overline{\mathrm{y}}$and $\overline{z}$ represent the mean of predisposing, enabling and need variables, μ is the mean of health service utilization variables, C_y_, C_j_ and C_k_ represent the CI of predisposing, enabling and need variables, and GC is the error term.

Horizontal inequity (HI) is the concentration index (CI) that is need-standardized health service utilization [19]. It reflects the level of inequality in health services use among people with equal needs. The need can be represented by need variables (self-assessed health status, chronic disease, disability, PADL and IADL). The HI is derived from the CI minus the contribution of the need variables, as follows:

$$ \mathrm{HI}=\mathrm{C}-{\sum}_{\mathrm{k}}\left(\frac{\upgamma_{\mathrm{k}}{\overline{\mathrm{z}}}_{\mathrm{k}}}{\upmu}\right){\mathrm{C}}_{\mathrm{k}}\ (4) $$

Similar to CI, HI ranges between [− 1,0), which indicates that the disproportionate distribution of health services utilization is more concentrated among the individuals with lower per capita household consumption expenditure and vice versa.

We explored the relevant influencing factors of health service utilization from the probability and frequency and decomposed the concentration index (CI) for the probability of health service utilization by the probit model and the frequency of health service utilization by the general negative binomial model.

All analyses were performed with the STATA 14.0 statistical software.

## Results

### Descriptive statistics

The social demographic characteristics of the participants are presented in Table 1. A total of 7836 respondents aged 60 and above were enrolled in the study. Approximately 20.94% of the elderly had received outpatient services in the last month, and the average number of outpatient visits was 0.47. About 17.14% of the respondents aged 60 and older had utilized inpatient health services at least once in the last year, and the average frequency of inpatient visits was 0.27. More than half of the elderly were generally satisfied with their health status, with 79.58% of the sampled participants reporting chronic diseases and 39.37% suffering from disabilities. Only 25.42% of the household members had disability in PADL and 37% of the respondents have disability in IADL. The majority of the elderly were enrolled in the NRCMS (64.77%), while approximately 11.24% of the participants were not covered by any health insurance scheme. Nearly half of the individuals (49.78%) were covered by the New Rural Pension (NRP), but 21.3% still did not participate in any pension insurance. There were more than half of the elderly from rural areas (60.95%), and 54.12% of the individuals were illiterate. Likewise, the majority of the elderly were working (56.24%) and married (82.21%). Finally, the proportion of the population in the eastern, central and western regions was relatively balanced. The comparison of demographic characteristics between the elderly using health services and those not using health services was detailed in Additional file 1: Tables SI and S2.

**Table 1:** Social demographic characteristics of the respondents, China, 2015

Variables	Category	All (*n* = 7836)
Dependent variables	At least one outpatient service in the last month, *n*(%)	1641(20.94)
	Frequency of outpatient services in the last month, mean (SD)	0.47(1.43)
	At least one inpatient service in the last year, *n*(%)	1343(17.14)
	Frequency of inpatient services in the last year, mean (SD)	0.27(0.74)
Predisposing variables		
Gender	Female^a^, *n*(%)	3850(49.13)
	Male, *n*(%)	3986(50.87)
Age	60~ 69^a^, *n*(%)	5279(67.37)
	70~ 79, *n*(%)	2154(27.49)
	80+, *n*(%)	403(5.14)
Education level	Illiterate^a^, *n*(%)	4241(54.12)
	Primary school, *n*(%)	1851(23.62)
	Middle school, *n*(%)	1147(14.64)
	High school and above, *n*(%)	597(7.62)
Marital status	Married, *n*(%)	6442(82.21)
	Unmarried^a^, *n*(%)	1394(17.79)
Employment status	Working, *n*(%)	4407(56.24)
	No^a^, *n*(%)	3429(43.76)
Enabling variables		
Health insurance schemes	No health insurance^a^, *n*(%)	881(11.24)
	UEBMI, *n*(%)	855(10.91)
	URBMI, *n*(%)	316(4.03)
	NRCMS, *n*(%)	5075(64.77)
	Other health insurance, *n*(%)	325(4.15)
	Two kinds of health insurance, *n*(%)	384(4.90)
Pension	No pension^a^, *n*(%)	1669(21.30)
	PPGI or BPIM, *n*(%)	851(10.86)
	NRPS, *n*(%)	3901(49.78)
	Old age pension allowance, *n*(%)	471(6.01)
	Other pension, *n*(%)	384(4.90)
	Two kinds of pension, *n*(%)	560(7.15)
Geographic location	East^a^, *n*(%)	2628(33.54)
	Central, *n*(%)	2577(32.89)
	West, *n*(%)	2631(33.58)
Residency location	Urban, *n*(%)	3060(39.05)
	Rural^a^, *n*(%)	4776(60.95)
Per capita household consumption expenditure	Quintile I(poorest)^a^, *n*(%)	1719(21.94)
	Quintile II, *n*(%)	1428(18.22)
	Quintile III, *n*(%)	1555(19.84)
	Quintile IV, *n*(%)	1567(20.00)
	Quintile V(richest), *n*(%)	1567(20.00)
Need variables		
Self-assessed health status	Health very good^a^, *n*(%)	788(10.06)
	Health good, *n*(%)	893(11.40)
	Health fair, *n*(%)	4120(52.58)
	Health poor, *n*(%)	1600(20.42)
	Health very poor, *n*(%)	435(5.55)
Chronic disease	Yes, *n*(%)	6236(79.58)
	No^a^, *n*(%)	1600(20.42)
Disability	Yes, *n*(%)	3085(39.37)
	No^a^, *n*(%)	4751(60.63)
PADL	Yes, *n*(%)	1992(25.42)
	No^a^, *n*(%)	5844(74.58)
IADL	Yes, *n*(%)	2899(37.00)
	No^a^, *n*(%)	4937(63.00)

Note: ^a^Reference group; UEBMI = Urban Employee Basic Medical Insurance; NRCMS = New Rural Cooperative Medical Scheme; URBMI = Urban Residents Basic Medical Insurance; PPGI or BPIM = Pension Program of the Government and Institutions or Basic Pension Insurance of the Firms; NRPS=New Rural Pension Scheme; After the sample of this study was divided into quintiles according to the per capita household consumption expenditure, the per capita household consumption expenditure of the five groups was in an ascending order of 0.33–500 yuan, 502–1250 yuan, 1254–2635 yuan, 2637–5675 yuan, and 5700–480,000 yuan, respectively

### Inequality and horizontal inequity for health services utilization

Table 2 reports CI and HI for probability and frequency of utilization of health services.

**Table 2:** Inequality and Horizontal Inequity for health services utilization, China, 2015

	Probability of Outpatient Visits	Frequency of Outpatient Visits	Probability of Inpatient Visits	Frequency of Inpatient Visits
Concentration index (CI)	0.1102	0.1015	0.2777	0.2980
Horizontal equity index (HI)	0.0899	0.0373	0.2544	0.1938

**Fig. 1:**
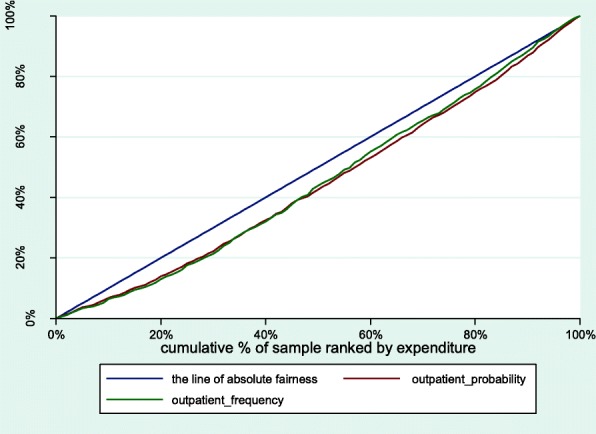
Concentration curves for use of outpatient services, China, 2015

The CI for the probability (CI = 0.1102) and the frequency (CI = 0.1015) of outpatient utilization were both positive, indicating that the better-off expenditure group had more advantages than the worse-off expenditure group in the probability and frequency of outpatient utilization (Fig. 1). After standardizing the differences in demands among the elderly, the HI for the probability (HI = 0.0899) and the frequency (HI = 0.0373) of outpatient utilization were lower than the corresponding CI, and there was the evidence of pro-rich inequity in outpatient utilization.

**Fig 2:**
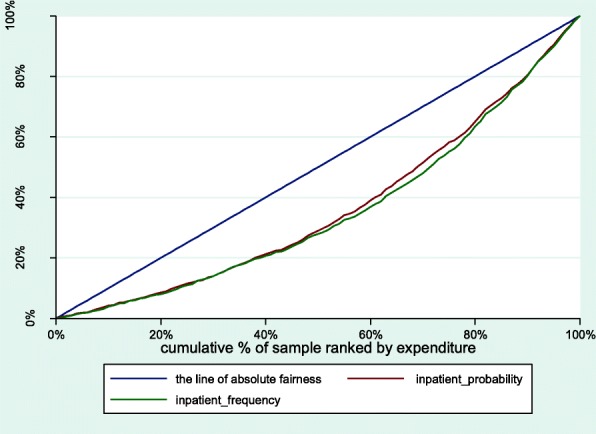
Concentration curves for use of inpatient services, China, 2015

Compared with outpatient utilization, the CI for the probability and the frequency of inpatient utilization were 0.2777 and 0.2980, respectively (Fig. 2). When controlling for need differences, the HI for the probability (HI = 0.2544) and the frequency (HI = 0.1938) of having inpatient service were also significantly lower than the CI for the related indicators.

### Decomposition of inequality in outpatient utilization

Table 3 shows the results of the decomposition of the CI for the outpatient service utilization among the elderly in China.

**Table 3:** Contribution to inequalities in utilization of outpatient service, China, 2015

Variable	Probability				Frequency		
	Elast^a^	Cont^b^	Percent		Elast^a^	Cont^b^	Percent
Predisposing variables							
Male	− 0.0448	0.00009	0.0826		− 0.0948	0.0002	0.1899
70~ 79	− 0.0147	0.0004	0.3520		− 0.0225	0.0006	0.5859
80+	0.0006	−0.00004	− 0.0358		− 0.0029	0.0002	0.1764
Primary school	0.0083	−0.00002	−0.0169		−0.0497	0.0001	0.1093
Middle school	0.0061	0.0005	0.4565		−0.0537	− 0.0044	−4.3365
High school and above	0.0125	0.0035	3.1981		−0.0022	−0.0006	− 0.6172
Married	−0.0101	− 0.00007	− 0.0620		− 0.0056	0.0000	−0.0374
Working	−0.0011	0.00008	0.0718		−0.0855	0.0064	6.3306
Enabling variables							
UEBMI	0.0317	0.0103	9.3594		0.0548	0.0178	17.5756
URBMI	0.0029	0.0003	0.2968		−0.0012	−0.0001	− 0.1299
NRCMS	0.1248	−0.0108	−9.8234		0.4192	−0.0363	−35.7979
Other health insurance	0.0179	0.0031	2.8319		0.0496	0.0087	8.5316
Two kinds of health insurance	0.0156	0.0032	2.9145		0.0518	0.0106	10.4741
PPGI or BPIM	0.0054	0.0016	1.4555		0.0323	0.0097	9.5238
NRPS	0.0299	−0.0027	−2.4484		0.0352	−0.0032	−3.1326
Old age pension allowance	0.0002	−0.00001	− 0.0077		− 0.0039	0.0002	0.1961
Other pension	−0.0025	−0.0002	− 0.2231		0.0214	0.0021	2.0863
Two kinds of pension	0.0054	0.0007	0.5905		0.0089	0.0011	1.0529
Central	−0.0303	0.0003	0.2312		−0.1236	0.0010	1.0244
West	0.0382	−0.0006	−0.5201		0.0691	−0.0010	−1.0193
Urban	−0.0493	−0.0065	−5.9027		−0.0661	− 0.0087	−8.5743
Quintile II	0.0440	−0.0167	−15.1406		0.1358	−0.0515	− 50.6898
Quintile III	0.0662	0.0001	0.0997		0.2086	0.0003	0.3409
Quintile IV	0.0720	0.0288	26.1225		0.1592	0.0637	62.7361
Quintile V (richest)	0.1032	0.0826	74.9192		0.2180	0.1744	171.7998
Need variables							
Health good	0.0068	−0.0002	−0.2252		0.0667	−0.0024	−2.4002
Health fair	0.2457	−0.0039	−3.5423		0.7741	−0.0123	−12.1159
Health poor	0.1913	0.0107	9.7519		0.5173	0.0291	28.6276
Health very poor	0.0612	0.0054	4.8685		0.1494	0.0131	12.8951
Chronic disease	0.3136	0.0092	8.3190		1.2659	0.0370	36.4488
Disability	0.0075	−0.0001	−0.1203		0.0252	− 0.0004	− 0.4407
PADL	0.0211	0.0006	0.5278		0.0967	0.0027	2.6204
IADL	0.0350	−0.0014	−1.2299		0.0676	−0.0026	−2.5829

Note: ^a^Elasticity (Elast); ^b^Contribution (Cont)

The level of per capital household consumption was the most important contributor to inequities in outpatient service use. Their contribution for the probability and the frequency of outpatient service use were 101.04% and 184.19%, respectively. The contribution of various insurance schemes to the inequity in outpatient services use showed a great difference. The UEBMI aggravated pro-rich inequality, and the decomposed values for probability and frequency of outpatient service utilization were 9.36% and 17.58%, respectively. On the contrary, the NRCMS made a negative contribution, and the corresponding decomposition results were − 9.82% and − 35.80%, respectively. The URBMI showed low contribution. Similar to the health insurance, the impact of different pension schemes on the utilization of outpatient services were also different. The PPGI and BPIM displayed a pro-rich impact on the outpatient service utilization, while the NRPS was opposite. Employment status of being working had a positive contribution to pro-rich inequality in outpatient use. But its contribution to probability of outpatient use (0.07%) was much lower relatively to that for the frequency of outpatient use (6.33%). Urban residency contributed to reducing pro-rich inequality in outpatient service use.

Among need variables, self-assessed health status of “health poor” and “health very poor” displayed a contribution in favor of the affluent, while “health good” and “health fair” were the opposite. The chronic diseases had a positive contribution to inequality for probability and frequency of outpatient use, and as high as 8.32% and 36.45%, respectively. The other variables provided relatively minor contribution to inequity. Other detailed data on the decomposition of inequality in outpatient use were shown in Additional file 1: Tables S3 and S4.

### Decomposition of inequality in inpatient utilization

Table 4 presents the contribution of various influencing factors to inequalities in inpatient service use.

**Table 4:** Contribution to inequalities in utilization of inpatient service, China, 2015

Variable	Probability				Frequency		
	Elast^a^	Cont^b^	Percent		Elast^a^	Cont^b^	Percent
Predisposing variables							
Male	0.0628	−0.0001	− 0.0464		0.2878	−0.0006	− 0.1964
70~ 79	0.0607	−0.0016	− 0.5821		0.2696	−0.0071	−2.3896
80+	0.0186	−0.0011	− 0.4133		0.0829	−0.0051	−1.7017
Primary school	0.0070	−0.00002	−0.0057		0.0025	0.0000	−0.0019
Middle school	0.0199	0.0018	0.5933		0.0552	0.0045	1.5183
High school and above	−0.0032	− 0.0009	− 0.3319		− 0.0327	− 0.0092	−3.1005
Married	− 0.0133	− 0.0001	− 0.0325		0.1729	0.0012	0.3910
Working	−0.1456	0.0110	3.9774		−0.6361	0.0478	16.0532
Enabling variables							
UEBMI	0.0316	0.0103	3.7325		0.1227	0.0399	13.4021
URBMI	0.0041	0.0005	0.1709		0.0145	0.0016	0.5510
NRCMS	0.0707	−0.0061	−2.2261		0.2026	−0.0176	−5.8940
Other health insurance	0.0043	0.0007	0.2698		0.0408	0.0071	2.3939
Two kinds of health insurance	0.0105	0.0022	0.7863		0.0409	0.0084	2.8188
PPGI or BPIM	−0.0215	− 0.0064	−2.3374		− 0.1322	− 0.0396	−13.2889
NRPS	0.0046	−0.0004	− 0.1519		−0.1449	0.0131	4.3951
Old age pension allowance	−0.0026	0.0001	0.0495		−0.0382	0.0020	0.6588
Other pension	0.0032	0.0003	0.1149		−0.0058	−0.0006	− 0.1940
Two kinds of pension	0.0068	0.0008	0.2987		−0.0352	−0.0042	−1.4142
Central	0.0407	− 0.0003	− 0.1244		0.1683	−0.0014	− 0.4751
West	0.0731	−0.0011	−0.3979		0.3131	−0.0047	−1.5744
Urban	0.0060	0.0008	0.2871		−0.0129	− 0.0017	− 0.5704
Quintile II	0.0437	−0.0166	−6.0173		0.2471	−0.0937	−31.4302
Quintile III	0.1137	0.0002	0.0685		0.4410	0.0007	0.2455
Quintile IV	0.1982	0.0793	28.8000		0.7599	0.3041	102.0304
Quintile V(richest)	0.2802	0.2242	81.4012		0.9770	0.7818	262.3329
Need variables							
Health good	0.0099	−0.0004	−0.1313		−0.0413	0.0015	0.5055
Health fair	0.1989	−0.0032	−1.1475		0.7266	−0.0115	−3.8746
Health poor	0.2127	0.0120	4.3396		0.7627	0.0428	14.3788
Health very poor	0.0778	0.0068	2.4754		0.2436	0.0213	7.1638
Chronic disease	0.2892	0.0085	3.0699		1.8373	0.0537	18.0216
Disability	0.0170	−0.0003	−0.1096		0.0685	−0.0012	−0.4077
PADL	0.0662	0.0018	0.6610		0.2797	0.0077	2.5827
IADL	0.0487	−0.0019	−0.6861		0.2600	−0.0101	−3.3826

Note: ^a^Elasticity (Elast); ^b^Contribution (Cont)

The per capital household consumption contributed as high as 104.25% and 332.68% to the inequity in probability and frequency of inpatient use. The contribution of health insurance varied according to the type of insurance schemes. The UEBMI had a positive contribution to the pro-rich inequity for probability (3.37%) and frequency (13.4%) of inpatient use. However, the corresponding contribution of NRCMS were − 2.23% and − 5.89%, which had a pro-poor impact on inpatient use. There were also great differences among a variety of pension schemes. The PPGI and BPIM contributed to increasing pro-rich inequality in inpatient use, while NRPS had the opposite effect. Urban residency, in the terms of probability and frequency of inpatient services use, alleviated the inequity. The employment status of being working, in the terms of probability and frequency of inpatient services use, exacerbated the inequity.

With respect to need variables, “health poor” and “health very poor” made a pro-rich contribution, while “health good” and “health fair” reduced the pro-rich inequality. In addition, the presence of chronic diseases made a great contribution to the increase of pro-rich inequality and other variables made little contribution to the change of inequality. Other detailed data on the decomposition of inequality in inpatient use were shown in Additional file 1: Tables S5 and S6.

## Discussion

This article analyzed the unfairness of the utilization of health services for the elderly using data from CHARLS. We identified that there was a pro-wealth inequality and horizontal inequity in the utilization of health services among elderly in China. It was consistent with the conclusions of the previous relevant scholars’ research [3, 20, 21]. However, MAO’s research on the utilization of health services for urban residents in western China showed that the probability and frequency of HI of outpatient services were − 0.004 and − 0.0116 respectively [22]. Li’s study reported that the probability and frequency of HI of health service utilization for middle-aged and old people in China were − 0.004 and − 0.0116, respectively [3]. In comparison, the overall unfairness reflected in this study was higher than that of previous studies, which may be caused by the differences between research objects and regions. Additionally, the data, stemming from Table 2, showed that the HI for the probability of utilization of health services accounted for the bulk of the overall inequity. The result suggested that patient-initiated effects dominate the inequity of health services utilization for the elderly, followed by doctors-driven.

More importantly, the paper revealed the contribution of various influencing factors to the inequity of health service use among the elderly in China. As Xie and Gong found, the greatest share of CI, deriving from contribution of per capita household expenditure to pro-rich inequality in both outpatient and inpatient use, demonstrated that the socioeconomic status was the most important factor influencing the utilization of health services [12, 23]. But as Rarick ‘s research showed, individuals with lower economic levels were associated with poorer self-rated health status, which means more demand for health services [24]. The contribution from per capita household consumption expenditures was clearly not conducive to equitable and rational utilization of health services. Therefore, effective measures must be taken to reduce the gap between the rich and the poor and to provide financial support to the elderly with lower economic levels.

It was well-known that health insurance schemes can help to decrease inequality in the utilization of health services [11, 25, 26]. Lei and Lin (2009) found that participating in the NRCMS significantly increased the use of preventive health care [27]. Zhou (2014) also identified both of UEBMI and URBMI reduced inequalities in the use of inpatient services [28]. However, Our study revealed that not all health insurance schemes were in line with the expected results. The health schemes such as UEBMI made a significantly contribution to increasing pro-rich inequity in health service use. Xie concluded that it was attributed to two main reasons: (1) The compensation rate for insured elderly of UEBMI was below the government’s expectation (75%) [29]. (2) The overuse of inpatient services by rich population may stimulated a greater focus of UEBMI on inpatient services, providing a higher reimbursement rate than other health insurance schemes [23]. In addition, another explanation for this result was that the “supplier-induced demand (SID)”, stemming from the doctor-driven effect, was more likely to occur in the insured elderly of UEBMI. This was also a good explanation for UEBMI’s pro-rich contribution to frequency of health services utilization (17.58% for outpatient and 13.40% for inpatient). To solve these problems, this paper puts forward the following specific suggestions: (1) The different health insurance schemes should be integrated and the reimbursement rate should be gradually raised to achieve the goal set by the Chinese government (75%). (2) The doctor’s performance-based pay system should be further improved so as to reflect the value of medical technical services.

In contrast, the result showed that NRCMS decreased the inequality for utilization of inpatient and outpatient. This was consistent with the results of previous studies [15, 20, 21, 23, 28]. This results can be explained by two reasons: (1) The Chinese government has raised the level of financing for the NRCMS, and the local government has raised the subsidy standards for the insured personnel of NRCMS. (2) The Chinese government has fully implemented the serious illness insurance system in all regions of the country. Nevertheless, the contributions of NRCMS on reducing unfairness in inpatient use were limited (− 2.23% for probability of inpatient use and − 5.89% for frequency of inpatient use). We think that the result is caused by the narrow benefit package of NRCMS in inpatient services. Therefore, it is necessary for the government to expand the welfare benefits of NCMS.

The presence of chronic diseases made a pro-rich contribution to inequality for outpatient use, which was significantly greater than its contribution in favor of rich to inequality for inpatient use. In China, four times health service surveys, conducted in 1998, 2003, 2008 and 2013, showed that the prevalence of chronic diseases in high-income groups was higher than in low-income groups [30–33]. Therefore, we insisted that the affluent elderly with chronic diseases have more outpatient services. In addition, the health insurance scheme provided inpatient-oriented welfare programs, resulting in less reimbursement rate for older persons seeking outpatient services for the treatment of chronic diseases. It increased the financial burden of patients with chronic diseases and further aggravated the pro-rich inequality in the utilization of outpatient services. In view of this problem, this study deems that the high priority chronic diseases should be included in basic public health services and the relevant interventions should be targeted to reduce inequalities caused by chronic diseases.

The employment status of being working increased the pro-rich contribution to the doctor-driven inequality for frequency of health services use (6.33% for outpatient and 16.05% for inpatient). Although no specific occupation type was involved in this study, this result corresponded to the findings of the UEBMI. This showed that the doctor-driven “SID” occurs mostly in working elderly. It could be explained that these people, having steady sources of income, brought more income to the doctors than unemployed individuals.

There are several limitations to this study. Firstly, the present study uses a cross-sectional data for analysis, which prevents us from discussing its findings based on causal relationships. Secondly, it has to be noted that some of the factors affecting the demand for health services were excluded from the regression model. For example, distance to the nearest health facilities is likely to affect the fairness of universal access to health services. However, the supply variables from the CHARLS cannot completely replace the factor, which makes it possible to ignore the key influencing factors in the discussion.

## Conclusion

In conclusion, there was a strong pro-rich inequality in both the probability and the frequency of use for health services among the elderly in China. The medical insurance schemes was not enough to solve these unfair problems, and different medical insurance schemes had different effects on the unfairness of health service utilization.

## Additional file

Additional file 1: **Table S1.** The comparison of demographic characteristics between the elderly using outpatient services and those not using outpatient services, China, 2015. **Table S2.** The comparison of demographic characteristics between the elderly using inpatient services and those not using inpatient services, China, 2015. **Table S3.** Contribution to inequalities in the probability of outpatient service utilization, China, 2015. **Table S4.** Contribution to inequalities in the frequency of outpatient service utilization, China, 2015. **Table S5.** Contribution to inequalities in the probability of inpatient service utilization, China, 2015. **Table S6.** Contribution to inequalities in the frequency of inpatient service utilization, China, 2015. (DOCX 54 kb)
