# Supplementary material for: Influencing factors of inequity in health services utilization among the elderly in China
Source: Int J Equity Health. 2018 Sep 15;17:144. doi: 10.1186/s12939-018-0861-6 (PMC6139169; doi:10.1186/s12939-018-0861-6)
Supplement: Supplementary file 1 — Table S1. The comparison of demographic characteristics between the elderly using outpatient services and those not using outpatient services, China, 2015. Table S2. The comparison of demographic characteristics between the elderly using inpatient services and those not using inpatient services, China, 2015. Table S3. Contribution to inequalities in the probability of outpatient service utilization, China, 2015. Table S4. Contribution to inequalities in the frequency of outpatient service utilization, China, 2015. Table S5. Contribution to inequalities in the probability of inpatient service utilization, China, 2015. Table S6. Contribution to inequalities in the frequency of inpatient service utilization, China, 2015. (DOCX 54 kb) [file 12939_2018_861_MOESM1_ESM.docx]

**Supplemental Table I** The comparison of demographic characteristics between the elderly using outpatient services and those not using outpatient services, China, 2015

| Variables | Category | Use  (n=1641) | Not use(n=6195) | χ2 | P |
| --- | --- | --- | --- | --- | --- |
| Dependent variables | Frequency of outpatient services in the last month, mean (SD) | 2.21(2.42) | — | — | — |
| Predisposing variables |  |  |  |  |  |
| Gender | Female^1^, n(%) | 862(52.53) | 2988(48.23) | 9.582 | <0.05 |
|  | Male, n(%) | 779(47.47) | 3207(51.77) |  |  |
| Age | 60~69^1^, n(%) | 1107(67.46) | 4172(67.34) | 0.008 | 0.996 |
|  | 70~79, n(%) | 450(27.42) | 1704(27.51) |  |  |
|  | 80+, n(%) | 84(5.12) | 319(5.15) |  |  |
| Education level | Illiterate^1^, n(%) | 894(54.48) | 3347(54.03) | 1.664 | 0.645 |
|  | Primary school, n(%) | 391(23.83) | 1460(23.57) |  |  |
|  | Middle school, n(%) | 225(13.71) | 922(14.88) |  |  |
|  | High school and above, n(%) | 131(7.98) | 466(7.52) |  |  |
| Marital status | Married, n(%) | 1334(81.29) | 5108(82.45) | 1.197 | 0.274 |
|  | Unmarried^1^, n(%) | 307(18.71) | 1087(17.55) |  |  |
| Employment status | Working, n(%) | 879(53.56) | 3528(56.95) | 6.038 | <0.05 |
|  | No^1^, n(%) | 762(46.44) | 2667(43.05) |  |  |
| Enabling variables |  |  |  |  |  |
| Health insurance schemes | No health insurance^1^, n(%) | 149(9.08) | 732(11.82) | 15.235 | <0.05 |
|  | UEBMI, n(%) | 187(11.40) | 668(10.78) |  |  |
|  | URBMI, n(%) | 53(3.23) | 263(4.25) |  |  |
|  | NRCMS, n(%) | 1088(66.37) | 3987(64.36) |  |  |
|  | Other health insurance, n(%) | 76(4.63) | 249(4.02) |  |  |
|  | Two kinds of health insurance, n(%) | 88(5.36) | 296(4.78) |  |  |
| Pension | No pension^1^, n(%) | 333(20.29) | 1336(21.57) | 4.278 | 0.51 |
|  | PPGI or BPIM, n(%) | 175(10.66) | 676(10.91) |  |  |
|  | NRPS, n(%) | 843(51.37) | 3058(49.36) |  |  |
|  | Old age pension allowance, n(%) | 93(5.67) | 378(6.10) |  |  |
|  | Other pension, n(%) | 72(4.39) | 312(5.04) |  |  |
|  | Two kinds of pension, n(%) | 125(7.62) | 435(7.02) |  |  |
| Geographic location | East^1^, n(%) | 519(31.63) | 2109(34.04) | 20.766 | <0.05 |
|  | Central, n(%) | 494(30.10) | 2083(33.62) |  |  |
|  | West, n(%) | 628(38.27) | 2003(32.33) |  |  |
| Residency location | Urban, n(%) | 595(36.26) | 2465(39.79) | 6.799 | <0.05 |
|  | Rural^1^, n(%) | 1046(63.74) | 3730(60.21) |  |  |
| Per capita household consumption expenditure | Quintile I(poorest)^1^, n(%) | 253(15.42) | 1466(23.66) | 72.818 | <0.05 |
|  | Quintile II, n(%) | 282(17.18) | 1146(18.50) |  |  |
|  | Quintile III, n(%) | 336(20.48) | 1219(19.68) |  |  |
|  | Quintile IV, n(%) | 357(21.76) | 1210(19.53) |  |  |
|  | Quintile V(richest), n(%) | 413(25.17) | 1154(18.63) |  |  |
| Need variables |  |  |  |  |  |
| Self-assessed health status | Health very good^1^, n(%) | 73(4.45) | 715(11.54) | 262.417 | <0.05 |
|  | Health good, n(%) | 102(6.22) | 791(12.77) |  |  |
|  | Health fair, n(%) | 820(49.97) | 3300(53.27) |  |  |
|  | Health poor, n(%) | 496(30.23) | 1104(17.82) |  |  |
|  | Health very poor, n(%) | 150(9.14) | 285(4.60) |  |  |
| Chronic disease | Yes, n(%) | 1473(89.75) | 4763(76.88) | 132.403 | <0.05 |
|  | No^1^, n(%) | 168(10.24) | 1432(23.12) |  |  |
| Disability | Yes, n(%) | 723(44.06) | 2362(38.13) | 19.119 | <0.05 |
|  | No^1^, n(%) | 918(55.94) | 3833(61.87) |  |  |
| PADL | Yes, n(%) | 541(32.97) | 1451(23.42) | 62.352 | <0.05 |
|  | No^1^, n(%) | 1100(67.03) | 4744(76.58) |  |  |
| IADL | Yes, n(%) | 722(44.00) | 2177(35.14) | 43.656 | <0.05 |
|  | No^1^, n(%) | 919(56.00) | 4018(64.86) |  |  |

Note: ^1^ Reference group;

**Supplemental Table II** The comparison of demographic characteristics between the elderly using inpatient services and those not using inpatient services, China, 2015

| Variables | Category | Use  (n=1343) | Not use  (n=6493) | χ2 | P |
| --- | --- | --- | --- | --- | --- |
| Dependent variables | Frequency of inpatient services in the last year, mean (SD) | 1.53(1.09) | — | — | — |
| Need variables |  |  |  |  |  |
| Gender | Female^1^, n(%) | 656(48.85) | 3194(49.19) | 0.053 | 0.818 |
|  | Male, n(%) | 687(51.15) | 3299(50.81) |  |  |
| Age | 60~69^1^, n(%) | 796(59.27) | 4483(69.04) | 49.611 | <0.05 |
|  | 70~79, n(%) | 453(33.73) | 1701(26.20) |  |  |
|  | 80+, n(%) | 94(7.00) | 309(4.76) |  |  |
| Education level | Illiterate^1^, n(%) | 705(52.49) | 3536(54.46) | 2.523 | 0.471 |
|  | Primary school, n(%) | 319(23.75) | 1532(23.59) |  |  |
|  | Middle school, n(%) | 212(15.79) | 935(14.40) |  |  |
|  | High school and above, n(%) | 107(7.97) | 490(7.55) |  |  |
| Marital status | Married, n(%) | 1069(79.60) | 5373(82.75) | 7.563 | <0.05 |
|  | Unmarried^1^, n(%) | 274(20.40) | 1120(17.25) |  |  |
| Employment status | Working, n(%) | 569(42.37) | 3838(59.11) | 126.741 | <0.05 |
|  | No^1^, n(%) | 774(57.63) | 2655(40.89) |  |  |
| Enabling variables |  |  |  |  |  |
| Health insurance schemes | No health insurance^1^, n(%) | 140(10.42) | 741(11.41) | 21.641 | <0.05 |
|  | UEBMI, n(%) | 187(13.92) | 668(10.29) |  |  |
|  | URBMI, n(%) | 58(4.32) | 258(3.97) |  |  |
|  | NRCMS, n(%) | 822(61.21) | 4253(65.50) |  |  |
|  | Other health insurance, n(%) | 56(4.17) | 269(4.14) |  |  |
|  | Two kinds of health insurance, n(%) | 80(5.96) | 304(4.68) |  |  |
| Pension | No pension^1^, n(%) | 294(21.89) | 1375(21.18) | 13.243 | <0.05 |
|  | PPGI or BPIM, n(%) | 147(10.95) | 704(10.84) |  |  |
|  | NRPS, n(%) | 624(46.46) | 3277(50.47) |  |  |
|  | Old age pension allowance, n(%) | 84(6.25) | 387(5.96) |  |  |
|  | Other pension, n(%) | 72(5.36) | 312(4.81) |  |  |
|  | Two kinds of pension, n(%) | 122(9.08) | 438(6.75) |  |  |
| Geographic location | East^1^, n(%) | 376(28.00) | 2252(34.68) | 25.056 | <0.05 |
|  | Central, n(%) | 456(33.95) | 2121(32.67) |  |  |
|  | West, n(%) | 511(38.05) | 2120(32.65) |  |  |
| Residency location | Urban, n(%) | 564(42.00) | 2496(38.44) | 5.906 | <0.05 |
|  | Rural^1^, n(%) | 779(58.00) | 3997(61.56) |  |  |
| Per capita household consumption expenditure | Quintile I(poorest)^1^, n(%) | 131(9.75) | 1588(24.46) | 362.763 | <0.05 |
|  | Quintile II, n(%) | 156(11.62) | 1272(19.59) |  |  |
|  | Quintile III, n(%) | 237(17.65) | 1318(20.30) |  |  |
|  | Quintile IV, n(%) | 350(26.06) | 1217(18.74) |  |  |
|  | Quintile V(richest), n(%) | 469(34.92) | 1098(16.91) |  |  |
| Need variables |  |  |  |  |  |
| Self-assessed health status | Health very good^1^, n(%) | 54(4.02) | 734(11.30) | 389.167 | <0.05 |
|  | Health good, n(%) | 80(5.96) | 813(12.52) |  |  |
|  | Health fair, n(%) | 589(43.86) | 3531(54.38) |  |  |
|  | Health poor, n(%) | 465(34.62) | 1135(17.48) |  |  |
|  | Health very poor, n(%) | 155(11.54) | 280(4.31) |  |  |
| Chronic disease | Yes, n(%) | 1231(91.66) | 5005(77.08) | 145.53 | <0.05 |
|  | No^1^, n(%) | 112(8.34) | 1488(22.92) |  |  |
| Disability | Yes, n(%) | 643(47.88) | 2442(37.61) | 49.154 | <0.05 |
|  | No^1^, n(%) | 700(52.12) | 4051(62.39) |  |  |
| PADL | Yes, n(%) | 535(39.84) | 1457(22.44) | 177.643 | <0.05 |
|  | No^1^, n(%) | 808(60.16) | 5036(77.56) |  |  |
| IADL | Yes, n(%) | 650(48.40) | 2249(34.64) | 90.418 | <0.05 |
|  | No^1^, n(%) | 693(51.60) | 4244(65.36) |  |  |

Note: ^1^ Reference group;

**Supplemental Table III** Contribution to inequalities in the probability of outpatient service utilization, China, 2015

| Variable | Partial effect | Std. Err. | Elasticity | Contribution | Percent |
| --- | --- | --- | --- | --- | --- |
| Need variables |  |  |  |  |  |
| Male | -0.0184 | 0.0098 | -0.0448 | 0.00009 | 0.0826 |
| 70~79 | -0.0112 | 0.0106 | -0.0147 | 0.0004 | 0.3520 |
| 80+ | 0.0026 | 0.0226 | 0.0006 | -0.00004 | -0.0358 |
| Health good | 0.0125 | 0.0241 | 0.0068 | -0.0002 | -0.2252 |
| Health fair | 0.0979** | 0.0184 | 0.2457 | -0.0039 | -3.5423 |
| Health poor | 0.1962** | 0.0258 | 0.1913 | 0.0107 | 9.7519 |
| Health very poor | 0.2310** | 0.0357 | 0.0612 | 0.0054 | 4.8685 |
| Chronic disease | 0.0825** | 0.0111 | 0.3136 | 0.0092 | 8.3190 |
| Disability | 0.0040 | 0.0099 | 0.0075 | -0.0001 | -0.1203 |
| PADL | 0.0174 | 0.0117 | 0.0211 | 0.0006 | 0.5278 |
| IADL | 0.0198 | 0.0111 | 0.0350 | -0.0014 | -1.2299 |
| Non-need variables |  |  |  |  |  |
| UEBMI | 0.0608* | 0.0253 | 0.0317 | 0.0103 | 9.3594 |
| URBMI | 0.0150 | 0.0302 | 0.0029 | 0.0003 | 0.2968 |
| NRCMS | 0.0404* | 0.0155 | 0.1248 | -0.0108 | -9.8234 |
| Other health insurance | 0.0902** | 0.0321 | 0.0179 | 0.0031 | 2.8319 |
| Two kinds of health insurance | 0.0668* | 0.0292 | 0.0156 | 0.0032 | 2.9145 |
| PPGI or BPIM | 0.0103 | 0.0198 | 0.0054 | 0.0016 | 1.4555 |
| NRPS | 0.0126 | 0.0130 | 0.0299 | -0.0027 | -2.4484 |
| Old age pension allowance | 0.0006 | 0.0217 | 0.0002 | -0.00001 | -0.0077 |
| Other pension | -0.0106 | 0.0230 | -0.0025 | -0.0002 | -0.2231 |
| Two kinds of pension | 0.0159 | 0.0207 | 0.0054 | 0.0007 | 0.5905 |
| Primary school | 0.0074 | 0.0118 | 0.0083 | -0.00002 | -0.0169 |
| Middle school | 0.0088 | 0.0149 | 0.0061 | 0.0005 | 0.4565 |
| High school and above | 0.0343 | 0.0212 | 0.0125 | 0.0035 | 3.1981 |
| Central | -0.0193 | 0.0112 | -0.0303 | 0.0003 | 0.2312 |
| West | 0.0239 | 0.0115 | 0.0382 | -0.0006 | -0.5201 |
| Urban | -0.0265* | 0.0110 | -0.0493 | -0.0065 | -5.9027 |
| Married | -0.0026 | 0.0124 | -0.0101 | -0.00007 | -0.0620 |
| Working | -0.0004 | 0.0104 | -0.0011 | 0.00008 | 0.0718 |
| Quintile II | 0.0506** | 0.0164 | 0.0440 | -0.0167 | -15.1406 |
| Quintile III | 0.0699** | 0.0164 | 0.0662 | 0.0001 | 0.0997 |
| Quintile IV | 0.0753** | 0.0165 | 0.0720 | 0.0288 | 26.1225 |
| Quintile V (richest) | 0.1081** | 0.0173 | 0.1032 | 0.0826 | 74.9192 |

Note: * p < 0.05; ** p < 0.01.

**Supplemental Table VI** Contribution to inequalities in the frequency of outpatient service utilization, China, 2015

| Variable | Partial effect | Std. Err. | Elasticity | Contribution | Percent |
| --- | --- | --- | --- | --- | --- |
| Need variables |  |  |  |  |  |
| Male | -0.0879 | 0.0711 | -0.0948 | 0.0002 | 0.1899 |
| 70~79 | -0.0386 | 0.0763 | -0.0225 | 0.0006 | 0.5859 |
| 80+ | -0.0269 | 0.1597 | -0.0029 | 0.0002 | 0.1764 |
| Health good | 0.2761 | 0.1915 | 0.0667 | -0.0024 | -2.4002 |
| Health fair | 0.6940** | 0.1481 | 0.7741 | -0.0123 | -12.1159 |
| Health poor | 1.1943** | 0.1611 | 0.5173 | 0.0291 | 28.6276 |
| Health very poor | 1.2686** | 0.1826 | 0.1494 | 0.0131 | 12.8951 |
| Chronic disease | 0.7499** | 0.0968 | 1.2659 | 0.0370 | 36.4488 |
| Disability | 0.0302 | 0.0680 | 0.0252 | -0.0004 | -0.4407 |
| PADL | 0.1793* | 0.0770 | 0.0967 | 0.0027 | 2.6204 |
| IADL | 0.0862 | 0.0757 | 0.0676 | -0.0026 | -2.5829 |
| Non-need variables |  |  |  |  |  |
| UEBMI | 0.2368 | 0.1686 | 0.0548 | 0.0178 | 17.5756 |
| URBMI | -0.0136 | 0.2081 | -0.0012 | -0.0001 | -0.1299 |
| NRCMS | 0.3051** | 0.1109 | 0.4192 | -0.0363 | -35.7979 |
| Other health insurance | 0.5635** | 0.1914 | 0.0496 | 0.0087 | 8.5316 |
| Two kinds of health insurance | 0.4981** | 0.1877 | 0.0518 | 0.0106 | 10.4741 |
| PPGI or BPIM | 0.1401 | 0.1393 | 0.0323 | 0.0097 | 9.5238 |
| NRPS | 0.0333 | 0.0891 | 0.0352 | -0.0032 | -3.1326 |
| Old age pension allowance | -0.0304 | 0.1635 | -0.0039 | 0.0002 | 0.1961 |
| Other pension | 0.2060 | 0.1809 | 0.0214 | 0.0021 | 2.0863 |
| Two kinds of pension | 0.0588 | 0.1525 | 0.0089 | 0.0011 | 1.0529 |
| Primary school | -0.0992 | 0.0805 | -0.0497 | 0.0001 | 0.1093 |
| Middle school | -0.1729 | 0.0979 | -0.0537 | -0.0044 | -4.3365 |
| High school and above | -0.0137 | 0.1364 | -0.0022 | -0.0006 | -0.6172 |
| Central | -0.1772* | 0.0868 | -0.1236 | 0.0010 | 1.0244 |
| West | 0.0970 | 0.0798 | 0.0691 | -0.0010 | -1.0193 |
| Urban | -0.0798 | 0.0829 | -0.0661 | -0.0087 | -8.5743 |
| Married | -0.0032 | 0.0912 | -0.0056 | 0.0000 | -0.0374 |
| Working | -0.0716 | 0.0717 | -0.0855 | 0.0064 | 6.3306 |
| Quintile II | 0.3512** | 0.1016 | 0.1358 | -0.0515 | -50.6898 |
| Quintile III | 0.4956** | 0.1094 | 0.2086 | 0.0003 | 0.3409 |
| Quintile IV | 0.3752** | 0.0999 | 0.1592 | 0.0637 | 62.7361 |
| Quintile V (richest) | 0.5139** | 0.1032 | 0.2180 | 0.1744 | 171.7998 |

Note: * p < 0.05; ** p < 0.01.

**Supplemental Table V** Contribution to inequalities in the probability of inpatient service utilization, China, 2015

| Variable | Partial effect | Std. Err. | Elasticity | Contribution | Percent |
| --- | --- | --- | --- | --- | --- |
| Need variables |  |  |  |  |  |
| Male | 0.0212* | 0.0086 | 0.0628 | -0.0001 | -0.0464 |
| 70~79 | 0.0378** | 0.0099 | 0.0607 | -0.0016 | -0.5821 |
| 80+ | 0.0620** | 0.0228 | 0.0186 | -0.0011 | -0.4133 |
| Health good | 0.0149 | 0.0224 | 0.0099 | -0.0004 | -0.1313 |
| Health fair | 0.0648** | 0.0169 | 0.1989 | -0.0032 | -1.1475 |
| Health poor | 0.1786** | 0.0257 | 0.2127 | 0.0120 | 4.3396 |
| Health very poor | 0.2402** | 0.0370 | 0.0778 | 0.0068 | 2.4754 |
| Chronic disease | 0.0623** | 0.0100 | 0.2892 | 0.0085 | 3.0699 |
| Disability | 0.0074 | 0.0087 | 0.0170 | -0.0003 | -0.1096 |
| PADL | 0.0446** | 0.0107 | 0.0662 | 0.0018 | 0.6610 |
| IADL | 0.0226* | 0.0099 | 0.0487 | -0.0019 | -0.6861 |
| Non-need variables |  |  |  |  |  |
| UEBMI | 0.0496* | 0.0219 | 0.0316 | 0.0103 | 3.7325 |
| URBMI | 0.0176 | 0.0258 | 0.0041 | 0.0005 | 0.1709 |
| NRCMS | 0.0187 | 0.0136 | 0.0707 | -0.0061 | -2.2261 |
| Other health insurance | 0.0176 | 0.0250 | 0.0043 | 0.0007 | 0.2698 |
| Two kinds of health insurance | 0.0369 | 0.0246 | 0.0105 | 0.0022 | 0.7863 |
| PPGI or BPIM | -0.0339* | 0.0143 | -0.0215 | -0.0064 | -2.3374 |
| NRPS | 0.0016 | 0.0114 | 0.0046 | -0.0004 | -0.1519 |
| Old age pension allowance | -0.0076 | 0.0181 | -0.0026 | 0.0001 | 0.0495 |
| Other pension | 0.0112 | 0.0211 | 0.0032 | 0.0003 | 0.1149 |
| Two kinds of pension | 0.0165 | 0.0179 | 0.0068 | 0.0008 | 0.2987 |
| Primary school | 0.0051 | 0.0104 | 0.0070 | -0.00002 | -0.0057 |
| Middle school | 0.0233 | 0.0135 | 0.0199 | 0.0018 | 0.5933 |
| High school and above | -0.0073 | 0.0165 | -0.0032 | -0.0009 | -0.3319 |
| Central | 0.0212* | 0.0105 | 0.0407 | -0.0003 | -0.1244 |
| West | 0.0373** | 0.0106 | 0.0731 | -0.0011 | -0.3979 |
| Urban | 0.0026 | 0.0098 | 0.0060 | 0.0008 | 0.2871 |
| Married | -0.0028 | 0.0107 | -0.0133 | -0.0001 | -0.0325 |
| Working | -0.0444** | 0.0092 | -0.1456 | 0.0110 | 3.9774 |
| Quintile II | 0.0411** | 0.0163 | 0.0437 | -0.0166 | -6.0173 |
| Quintile III | 0.0982** | 0.0172 | 0.1137 | 0.0002 | 0.0685 |
| Quintile IV | 0.1699** | 0.0184 | 0.1982 | 0.0793 | 28.8000 |
| Quintile V (richest) | 0.2401** | 0.0195 | 0.2802 | 0.2242 | 81.4012 |

Note: * p < 0.05; ** p < 0.01.

**Supplemental Table VI** Contribution to inequalities in the frequency of inpatient service utilization, China, 2015

| Variable | Partial effect | Std. Err. | Elasticity | Contribution | Percent |
| --- | --- | --- | --- | --- | --- |
| Need variables |  |  |  |  |  |
| Male | 0.1520* | 0.0632 | 0.2878 | -0.0006 | -0.1964 |
| 70~79 | 0.2636** | 0.0657 | 0.2696 | -0.0071 | -2.3896 |
| 80+ | 0.4334** | 0.1279 | 0.0829 | -0.0051 | -1.7017 |
| Health good | -0.0973 | 0.1847 | -0.0413 | 0.0015 | 0.5055 |
| Health fair | 0.3714* | 0.1621 | 0.7266 | -0.0115 | -3.8746 |
| Health poor | 1.0039** | 0.1670 | 0.7627 | 0.0428 | 14.3788 |
| Health very poor | 1.1794** | 0.1820 | 0.2436 | 0.0213 | 7.1638 |
| Chronic disease | 0.6205** | 0.1302 | 1.8373 | 0.0537 | 18.0216 |
| Disability | 0.0467 | 0.0695 | 0.0685 | -0.0012 | -0.4077 |
| PADL | 0.2957** | 0.0701 | 0.2797 | 0.0077 | 2.5827 |
| IADL | 0.1889* | 0.0745 | 0.2600 | -0.0101 | -3.3826 |
| Non-need variables |  |  |  |  |  |
| UEBMI | 0.3022* | 0.1282 | 0.1227 | 0.0399 | 13.4021 |
| URBMI | 0.0965 | 0.1729 | 0.0145 | 0.0016 | 0.5510 |
| NRCMS | 0.0841 | 0.1036 | 0.2026 | -0.0176 | -5.8940 |
| Other health insurance | 0.2646 | 0.1613 | 0.0408 | 0.0071 | 2.3939 |
| Two kinds of health insurance | 0.2243 | 0.1470 | 0.0409 | 0.0084 | 2.8188 |
| PPGI or BPIM | -0.3271** | 0.1181 | -0.1322 | -0.0396 | -13.2889 |
| NRPS | -0.0782 | 0.1058 | -0.1449 | 0.0131 | 4.3951 |
| Old age pension allowance | -0.1706 | 0.1394 | -0.0382 | 0.0020 | 0.6588 |
| Other pension | -0.0321 | 0.1427 | -0.0058 | -0.0006 | -0.1940 |
| Two kinds of pension | -0.1322 | 0.1177 | -0.0352 | -0.0042 | -1.4142 |
| Primary school | 0.0029 | 0.0752 | 0.0025 | 0.0000 | -0.0019 |
| Middle school | 0.1013 | 0.0866 | 0.0552 | 0.0045 | 1.5183 |
| High school and above | -0.1154 | 0.1244 | -0.0327 | -0.0092 | -3.1005 |
| Central | 0.1376 | 0.0772 | 0.1683 | -0.0014 | -0.4751 |
| West | 0.2506* | 0.0784 | 0.3131 | -0.0047 | -1.5744 |
| Urban | -0.0089 | 0.0714 | -0.0129 | -0.0017 | -0.5704 |
| Married | 0.0565 | 0.0783 | 0.1729 | 0.0012 | 0.3910 |
| Working | -0.3040** | 0.0672 | -0.6361 | 0.0478 | 16.0532 |
| Quintile II | 0.3644** | 0.1316 | 0.2471 | -0.0937 | -31.4302 |
| Quintile III | 0.5972** | 0.1118 | 0.4410 | 0.0007 | 0.2455 |
| Quintile IV | 1.0213** | 0.1120 | 0.7599 | 0.3041 | 102.0304 |
| Quintile V (richest) | 1.3131** | 0.1051 | 0.9770 | 0.7818 | 262.3329 |

Note: * p < 0.05; ** p < 0.01.
